# Dementia in-patient units in psychiatric hospitals: research priority setting

**DOI:** 10.1192/bjb.2024.42

**Published:** 2025-08

**Authors:** George Crowther, Rebecca Dunning, Gregor Russell, Emma Wolverson, Benjamin R. Underwood

**Affiliations:** 1 Leeds and York Partnership NHS Foundation Trust, Leeds, UK; 2 Leeds Institute of Health Science, University of Leeds, UK; 3 Humber Teaching NHS Foundation Trust, Hull, UK; 4 Bradford District Care NHS Foundation Trust, Saltaire, UK; 5 Faculty of Health Sciences, University of Hull, UK; 6 Dementia UK, London, UK; 7 Cambridgeshire and Peterborough NHS Foundation Trust, Cambridge, UK; 8 Department of Psychiatry, University of Cambridge, UK

**Keywords:** Dementias/neurodegenerative diseases, in-patient treatment, dementia in-patient unit, research priorities, Delphi

## Abstract

**Aims and method:**

Dementia in-patient units (DIU) are mental health wards that care for people living with dementia (PLWD) whose symptoms are causing severe distress or potential risk. DIUs look after some of the most vulnerable and unwell people in society, yet they are environments that are underresearched: a recent systematic review revealed only 36 articles worldwide relating to DIUs. To better understand research priorities in DIUs, we undertook a two-round online Delphi survey of PLWD with experience of DIUs, their carers and professionals who work in DIUs.

**Results:**

Ten research priorities were described and ranked. The top three were how to use non-pharmacological techniques to manage non-cognitive symptoms of dementia, supporting families and better understanding of how to discharge PLWD safely and healthily.

**Clinical implications:**

This is the first Delphi consensus to describe DIU research priorities. This paper will help researchers focus on the areas that matter most to people who use DIUs.

There are around 55 million people living with dementia (PLWD) worldwide, and 900 000 PLWD in the UK.^1,2^ Severe psychological symptoms in dementia can cause observed behaviour such as agitation, panic, anger and social withdrawal. Collectively, these symptoms are termed behavioural and psychological symptoms of depression (BPSD).^3^ In situations where distress-related behaviour cannot be safely managed at an individual's normal place of residence, they might be admitted to a specialist mental health ward in a psychiatric hospital, called dementia in-patient units (DIUs). Commonly cited distress-related behaviours that lead to admission are aggression, sexual disinhibition, self-neglect, euphoria, agitation and wandering.^4–12^ It is estimated there are more than 50 of these wards in the UK, although an exact number is unknown. DIUs in mainland Europe, North America, Australasia, Africa and Asia are also descried in the literature.^13^

DIUs are usually staffed by a combination of mental health nurses, psychiatrists (a mixture of consultants, speciality doctors and doctors in training), healthcare assistants, speech and language therapists, occupational therapists and physiotherapists.^14^ Some may also have music and art therapists and psychologists.

The focus of most admissions is to reduce distress-related behaviours and promote discharge to a less restrictive environment; however, it is not immediately clear what constitutes a ‘good outcome’ for this patient group or how to achieve it. This is because of a dearth of evidence-based literature available to advise on best practice. A recent systematic review by Wolverson et al^13^ found only 36 articles worldwide that described the characteristics, care and outcomes of DIUs. Of the studies retrieved, 33 were audits. They described a population of men and women, with predominantly Alzheimer's type dementia of varying severities.^13^ They are a group with high levels of medical comorbidity^15^ and mortality; with around 8% of patients dying during admission^14^ and a further 5.9% of people dying within 3 months of discharge.^16^

Given the relative paucity of research in DIUs, and to ensure that future research has the greatest impact for patients and future research investment, we sought to establish a list of research priorities.

## Aim

We aimed to define the research priorities for DIUs from the perspective of people who have used them, their carers and the healthcare professionals (HCPs) that work within them.

## Method

### Data collection

We undertook a two-round online Delphi survey with PLWD with experience of in-patient mental healthcare, their family carers and professionals who work in these environments. A Delphi methodology was selected because it is a well-described and accepted method of collecting opinions about real-world issues from a group with experience in the chosen field.^17^

In round one, two separate meetings were held, one with HCPs and one with PLWD and their carers. Separate meetings helped to ensure that the voices of both groups were heard and that one did not overshadow the other. Attendees were given a short overview/introduction by the authors describing the current limited evidence base. Data for this presentation were taken from a systematic review on research outputs from DIUs conducted by this team.^13^ Participants were asked to list freehand, and without consultation with others, their top five dementia in-patient research priorities. The online survey platform SurveyMonkey (www.surveymonkey.com) was used to collect results. Answers were anonymous.

All participants from round one were invited to participate in round two ,which took place 5 months later. Again, two separate meetings were held, one with PLWD and their carers and one with HCPs. Attendees were given a short presentation in which the top ten research priority areas from round one were presented in a random order, along with a description of each priority (see Table 1). Participants were asked to anonymously and independently rank them in order of preference, using the SurveyMonkey platform (www.surveymonkey.com).

**Table 1:** Content analysis results

Theme	Explanation of theme	Response numbers (total)
Non-pharmacological management of BPSD	What strategies and care approaches (things other than medications) help people with dementia when they are very distressed on DIUs?	34
Understanding in-patient wards	To learn more about why people living with dementia are admitted to DIUs, how long people stay and where they go after being in hospital, what other health problems people have, how many people are dying on DIUs and how common adverse incidents like falls happen.	20
Pharmacological management of BPSD	What are the most useful medications to help people with dementia when they are experiencing distressing symptoms?	16
Ward safety	How can we reduce the use of seclusion and restraint in people living with dementia admitted to DIUs?	12
Physical health management	Developing a better understanding of the physical healthcare needs of people living with dementia who are in hospital, and how to access appropriate treatment for them.	11
Staff well-being	Improving staff well-being and psychological safety at work.	12
Family support	What are the needs of families and carers of people living with dementia admitted to a DIU?	10
End-of-life care	To learn more about the needs of people with dementia, families and staff who come to the end of their life on DIUs.	8
Transitions of care	Improving the interface of DIUs with community care.	7
Developing national ward networks	Creating a national database of all available wards and beds.	1

BPSD, behavioural and psychological symptoms of depression; DIU, dementia in-patient unit.

Ethical approval was not sought for the work as it fell within the realm of patient and public engagement in research, rather than primary research.

#### Sample

We aimed to get opinions from experts in the field of dementia in-patient care, including HCPs, PLWD who have experience of DIUs and carers of people with dementia who been cared for in DIUs. The sample size was dependant on group dynamics and ability to reflect the population the population using or benefiting from the research, rather than a predetermined number.^18^

### HCPs

A group of HCPs with extensive clinical experience in the field was established, using expert and snowball sampling. Expressions of interest to join the DIU expert group were invited from Members of the Royal College of Psychiatrists Faculty of Old Age Psychiatry via email and at their annual conference during a lecture hosted by the authors (approximately *n* = 200). Purposive criterion sampling was used to ensure that HCPs from a range of clinical backgrounds with DIU experience attended, including psychiatry, psychology, occupational therapy, elderly medicine, art and music therapy, pharmacy and nursing.

### PLWD and their carers

In round one, an established specialist in-patient patient and public involvement and engagement (PPIE) group of people with dementia and their carers with experience of mental health in-patient care was invited to take part in the survey. The PPIE group was established to support dementia research. The group meet monthly online, and was chaired by one of the research team (E.W.). In round two, the PPIE group was a approached again, and the link to the survey was also shared on social media to try to engage more people with dementia and their families.

To ensure PLWD had a good understanding of the research, materials used plain language to communicate with individuals with dementia, avoiding any jargon or ambiguous terms. The team recognised that DIUs can be given different names (mental health wards, psychiatric wards or units), and that this may cause confusion. Care was taken in the first meeting to describe the setting and how it differed from care homes and acute hospitals. Ground rules were set at the start of the PLWD and carer meeting, including an agreement to allow members with dementia to speak first and without delay, to ensure that people did not forget what they wanted to say. People with dementia and their carers were offered the opportunity to receive a paper version of the survey by post, but nobody chose this option. Surveys were kept short, and instructions were simple. Support was offered (via telephone or teams meeting) to complete the survey with a researcher, but nobody requested support.

### Analysis

#### Round one

In round one, participants listed up to five research priorities each. No weighting on order of preference was placed on these responses. This produced a freehand list. Content analysis was performed and responses were grouped into themes and ranked by the number of responses given to each theme.^19^ Where there were disagreements between the researchers, consensus from the wider group was sought. This resulted in ten priorities.

Content analysis was selected as it is recommended for use when there is limited knowledge about a subject or when the knowledge is fragmented.^20^ One advantages of content analysis is that the data from participants drives the generation of codes and interpretation, rather than any preconceived categories or theoretical perspectives.^21^

The authors (G.C. and R.D.) independently conducted the analysis. G.C. is a male liaison psychiatrist and R.D. is a female assistant psychologist. Both have experiences of working within DIUs. Both G.C. and R.D. have experience of conducting qualitative data analysis. The entire data were discussed in research meetings with all authors, and all authors were involved in the process of constructing final themes. The wider team is made up of both psychologists and psychiatrists, all with experience of clinical work and leadership within DIUs. The team were frustrated by the lack of research into in-patient care to guide practice, and felt that priorities would provide some guidance.

#### Round two

The two groups independently ranked the ten priorities from 1 (most important) to 10 (least important). For each group (PPIE and HCP), the total score for each research priority was then added together. A Borda count voting system was employed to rank the priorities. The priority theme with the lowest score is ranked most important in this system.^22^

The PPIE group had fewer attendees than the HCP group. To ensure the PPIE group priorities were given equal weighting to HCP group priorities, the scores from the PPIE group were multiplied by a factor of the difference between group sizes.

## Results

### Participants

In round one, two separate meetings were held; one for PLWD and their carers and one for HCPs. One person living with dementia, eight carers and 30 HCPs completed round one. Everyone supplied at least one priority; the maximum was five.

In round two, one meeting was held with HCPs, and two meetings were held with PLWD and their carers. Two PLWD, 11 carers and 47 HCPs completed round two. The breakdown of participants by their relationship with dementia can be found in Table 2.

**Table 2:** Participant breakdown

Role	Round 1	Round 2
Person living with dementia	1	2
Carer of someone living with dementia	8	11
Psychiatrist	10	11
Psychologist	4	8
Nurse	10	23
Geriatrician	1	2
Pharmacist	2	0
Music therapist	1	1
Art therapist	1	0
Occupational therapist	1	1
Speech and language therapist	0	1
Dementia charity organisation	1	0
Total	40	60

### Round one

The research priority theme with the most codes was the ‘non-pharmacological management of BPSD’ (31 codes). Although most responses were non-specific, some suggested that the use of activity and engagement, use of alternative and holistic therapies, use of exercise and outdoor spaces, sleep hygiene and psychological interventions should be priorities. One respondent suggested researching effective communication strategies in dementia.

The next most popular theme was ‘understanding DIUs’ (20 codes). The most common response within this theme focused on improving understanding of reasons for admission; this included demographic information about patients and their circumstances, and understanding the potential role of social care and community services in preventing admission. Some respondents questioned whether admissions worked, and the adverse effects of admission. Numerous responses questioned whether admissions are always appropriate. Other responses suggested focusing on the length of stay at DIUs. Discharge was commonly suggested as an area of interest. This included understanding how to better support discharge home, avoiding readmission and the impact of delayed discharges resulting from placement unavailability.

Sixteen codes related to exploring the ‘pharmacological management of BPSD’. Most responses within this theme focused on polypharmacy and deprescribing. Other responses included focus specifically on the use of antipsychotics and sedation.

Research priorities relating to ‘ward safety’ (12 codes) were the fourth most common. Within this theme, understanding and reducing restrictive interventions was considered a research priority by many respondents. This included understanding the use of restrictive interventions, incidents of safeguarding and causes of adverse incidents. Another priority for respondents was understanding assaults on both staff and peers. Other respondents highlighted better understanding of falls within this category.

There were three main subthemes within the ‘staff well-being’ theme (12 codes). Some respondents spoke about better understanding of the staffing levels and mix of professions on in-patient wards. Others spoke about offering staff support through improving their psychological safety, promoting well-being and offering reflective spaces. Respondents also suggested hearing staff views on their roles, and upskilling them with further training.

Eleven codes related to ‘better understanding of physical healthcare’ on DIUs. This included investigating treatable comorbidities and access to other specialist clinicians. Some questioned access to neuroimaging for DIUs. One respondent specifically highlighted the need to understand incontinence support available to DIUs, and one suggested investigating the long-term effects of COVID-19 on in-patient wards.

The theme ‘family support’ had ten codes relating to it, reflecting the varied responses within this theme. They included trying to identify the needs of families and the support they receive during admissions. Some respondents referred to ways to involve families in the care of a loved one. Two respondents recognised the ongoing needs of families, with one mentioning preparing families for end of life and another mentioning preparing them for transitions into care.

There were eight responses coded into the theme ‘end-of-life care’. Most respondents within this theme identified wanting to develop the understanding of end-of-life care on DIUs, with one respondent specifically mentioning understanding and managing end-of-life agitation.

Seven responses were coded into ‘transitions of care to the community’ theme. Specifically, within this theme respondents wanted to better understand how to safely and effectively move PLWD back to their home or care home with minimal distress.

Finally, one respondent felt that resources should be put into developing a national network of DIUs to create a database of outcome data, and allow other Trusts to know where available beds are around the country.

### Round two

Two PLWD, 11 carers and 47 HCPs took part in round two. HCPs outnumbered PLWD and their carers by a factor of 4.62, and aggregated scores from this group were multiplied accordingly to provide equal weighting. A Borda count of was performed. Results ordered by ranking are displayed in Table 3. Non-pharmacological management of BPSD was the most popular research priority. Pharmacological management of BPSD and developing national ward networks were the least popular research priority.

**Table 3:** Dementia in-patient unit research priorities

Research priority	Patient and carer score	Weighted patient and carer score (×4.62)	Healthcare professional score	Total	Ranking
Non-pharmacological management of behavioural and psychological symptoms of dementia	42	194.0	138	332.0	1
Family support	54	249.5	232	481.5	2
Understanding in-patient wards	57	263.3	229	492.3	3
Transitions of care	60	277.2	286	563.2	4
Staff well-being	84	388.1	213	601.1	5
Ward safety	86	397.3	208	605.3	6
End-of-life care	67	309.5	298	607.5	7
Physical health management	85	392.7	234	626.7	8
Pharmacological management of behavioural and psychological symptoms of dementia	91	420.4	257	677.4	9
Developing national ward networks	83	383.5	347	730.5	10

## Discussion

DIUs exist throughout the world, and look after the most vulnerable and unwell people with dementia in our society. Yet, they are environments that are underresearched, poorly understood and have not seen advances in models of care delivery that have benefitted equivalent specialist environments in healthcare, such as specialist oncology, cardiac or stroke units. This study is the first to describe research priorities in dementia in-patient care. All ten research priorities listed are important, and represent areas that have a limited evidence base to inform best practice.

Developing and testing ways of managing BPSD using non-pharmacological techniques was the highest priority for PLWD, their carers and HCPs in both rounds. Managing BPSD should be person-centred and holistic, with the aim of improving quality of life.^23,24^ The realities of a busy ward environment – caring for people with distress, disinhibition, social withdrawal and, at times, anger – makes delivering this challenging. The default is then a pharmacological option. Despite there being no available evidence about the effectiveness of medications in this environment, adverse drug outcomes or how to design personalised medication plans, Rouch et al^16^ describe patients being discharged from a DIU with an average of 2.28 different psychotropics each. The most commonly used medication types are antipsychotics and benzodiazepines.^14,16^ The side effects can be serious and are well described, such as falls, stroke and cardiac arrhythmias. Each decision to prescribe is a balancing act, with limited evidence to inform decisions. What little guidance there is available suggests that a non-pharmacological approach should be adopted, at least initially. However, the evidence base for what is effective in this environment is limited to music therapy,^25^ which is not universally available and is not an acute intervention. In an acute situation where symptoms are so severe as to cause imminent risk to the PLWD, staff or other patients, there are few practical options available to manage the situation other than verbal de-escalation, restraint and reasoned medication delivery. This is perhaps why pharmacological management was prioritised higher among HCPs than PLWD and their carers. In care homes, which are similar environments to DIUs, the evidence base for non-pharmacological management of BPSD is far better developed.^26^ This raises the question as to whether these interventions would ‘reach across’ into DIUs in the future.

DIUs are usually staffed by a multidisciplinary team of HCPs.^14^ A large number of shifts are covered by temporary staff.^14^ Knowing how to better support family and staff on dementia mental health wards was important to all respondents, but understandably more so for carers. The experience of being on a DIU can be frightening for both the PLWD and their carers, who often feel uninformed and unheard.^13^ They should have a loud voice in all aspects of care and future research.

Ward safety (reducing restraint, violent incidents and falls) was important to all groups, but more so to HCPs. When such events happen, they have direct implications for the PLWD and their carers, but can also cause moral injury to staff, which is further compounded by the subsequent investigations that follow. This high level of stress has been suggested as a possible cause for the high levels of staff sickness and agency nursing on these wards.^14^ Working to reduce these incidents may have benefits to both ward safety and staff welfare (priority 6).

Managing physical healthcare in mental health is a national research priority,^27^ but is usually targeted at those with severe functional mental illness. There is undoubtedly a need in this group, but those with dementia should not be neglected; people with dementia have high levels of comorbidity, and dementia is the leading cause of death in the UK and a common cause of death on DIUs.^14,28^ It is crucial that DIUs know how to manage patients’ physical health, which will include liaising with elderly medicine and palliative care services (priorities 7 and 8).

A hospital admission should be an interlude in someone's dementia journey, not the destination. Once a decision has been made to admit a PLWD to a DIU, they need an available bed, and a plan in place as soon as possible as to how to safely discharge them back to the community once they are well enough. In the UK, the current system has bed occupancy near 100%,^14^ with no clear system for communicating national bed availability. Furthermore, residential and care homes are near capacity, causing delays to discharges even when they are psychiatrically well.^29^ We need to understand how to improve transitions of care (priority 4), both onto and off of DIUs. This may entail developing a network of wards, to create a national database of wards and beds (priority 10). Other areas of medicine, such as intensive care, maternity and neonatal care, all have national bed coordination to ensure that specialist in-patient care can be delivered in a timely fashion when needed.^30^ These systems also allow for interdepartmental outcome benchmarking, helping units to learn from one another's successes and recognising if they have areas they can improve on. Understanding what factors predict a successful discharge in a timely fashion could help reduce length of stay and readmission rates.

### Limitations

People who are admitted to a mental health ward because of their dementia symptoms often have severe cognitive impairment. This limits the number of people available to contribute to our PPIE group; only three PLWD participated in the study (one in round 1 and two in round 2). Carers were more numerous (eight in round 1 and 11 in round two), but HCPs still outnumbered the PPIE group by 4.6:1. This was accounted for by weighting the responses from the PPIE group. There is relatively good congruence between HCPs and PLWD and their carers. This reduces concerns that the large multiplier on the latter's score has affected the findings, and gives confidence that there is an agenda shared by all participants as to where research priorities should focus.

There was a 5 month gap between round one and round two. There were no significant changes in national dementia in-patient policy or published research findings in this time that would have influenced the results. In this time, both the HCP and PPIE groups increased in number and changed slightly, although membership criteria did not alter. Priorities between rounds one and two are largely similar, other than pharmacological treatments of BPSD (which moved from position 3 to 9). This consistency reduces the concern that this gap affected the findings.

The iterative nature of Delphi studies can enable investigators to mould participant opinion.^17^ A presentation of round one results was given at the start of round two. The investigators made every effort to deliver the priorities in an unbiased fashion. Priorities were presented in a random order, and round two participants were not made aware of which priority was the most popular in round one. Furthermore, during round two ranking, the list of potential priorities shown to participants was randomly ordered.

To conclude, dementia research is a national priority.^31^ Although only a small proportion of PLWD require in-patient care, they are some of the most unwell (physically and mentally) and vulnerable patients in the whole of healthcare. We would support advances in all of the listed priorities, but advocate for the role of PLWD, their carers and the HCPs that look after them in championing research into non-pharmacological management of BPSD, supporting families and staff, and better understanding of DIUs and how to support PLWD to be discharged from them safely and healthily.

## Data Availability

The data that support the findings of this study are available from the corresponding author, G.C., on reasonable request.

## About the authors

**George Crowther** is a consultant in old age liaison psychiatry at Leeds and York Partnership NHS Foundation Trust, Leeds, UK, and a visiting senior lecturer at Leeds Institute of Health Science, University of Leeds, UK. **Rebecca Dunning** is an assistant psychologist at Humber Teaching NHS Foundation Trust, Hull, UK. **Gregor Russell** is a visiting associate professor at Leeds Institute of Health Science, University of Leeds, UK, and a consultant old age psychiatrist at Bradford District Care NHS Foundation Trust, Saltaire, UK. **Emma Wolverson** is a reader in ageing and dementia at the University of Hull, UK, and research lead for Dementia UK, London, UK. **Benjamin R. Underwood** is an honorary consultant psychiatrist at Cambridgeshire and Peterborough NHS Foundation Trust, Cambridge, UK, and an assistant professor in the Department of Psychiatry, University of Cambridge, UK.
